# SURROGATE HEALTH INFORMATION-SEEKING FOR OLDER ADULTS: INTERGENERATIONAL SUPPORT IN LIFE TRANSITION CONTEXTS

**DOI:** 10.1093/geroni/igad104.0249

**Published:** 2023-12-21

## Abstract

Due to a lack of experience with technology, many older Chinese rely on their younger family members to search for online health information. This study draws on life transition theory to explore the impact of family-bonded intergenerational support on surrogate health information-seeking (SHIS) during COVID-19. A semi-structured interview was conducted with 28 participants (Mean=32.45, SD=2.36) who had SHIS experiences with their older family members. Data were analyzed using qualitative thematic analysis. The results suggested that intergenerational support in SHIS during COVID-19 included: (1) provision of preventive and diagnostic health information, (2) provision of information on epidemic prevention policies and consultation, (3) guidance on the use of social media and Internet applications for older adults, and (4) communication of emotional support and hedonic information on daily life. Furthermore, intergenerational support in SHIS helps to promote reconstructed health information practices of older adults in life transitions and refine elders’ meaning-making.

